# Plasma Lipidomics and Proteomics Analyses Pre- and Post-5000 m Race in Yili Horses

**DOI:** 10.3390/ani15070994

**Published:** 2025-03-30

**Authors:** Jianwen Wang, Wanlu Ren, Zexu Li, Luling Li, Ran Wang, Shikun Ma, Yaqi Zeng, Jun Meng, Xinkui Yao

**Affiliations:** 1 College of Animal Science, Xinjiang Agricultural University, Urumqi 830052, China; wjw1262022@126.com (J.W.); 13201295117@163.com (W.R.); 13593312012@163.com (Z.L.); 18996888638@163.com (L.L.); 17590811761@163.com (R.W.); 18299152719@163.com (S.M.); xjauzengyaqi@163.com (Y.Z.); 2 Xinjiang Key Laboratory of Equine Breeding and Exercise Physiology, Urumqi 830052, China

**Keywords:** differential lipids, differentially expressed proteins, metabolism

## Abstract

The impact of exercise on human metabolism has been extensively studied, yet limited research exists on the effects of high-intensity racing on equine metabolism. The aim of this study was to screen the effect of a 5000 m race on lipids and proteins in the plasma of Yili horses for the breeding of racehorses. Blood samples were collected from the top three finishers, and lipidomics and proteomics analyses were performed. Lipidomic analysis identified 10 differential lipids. Compared to pre-race levels, phosphatidylethanolamine (18:0/16:0) (PE (18:0/16:0)) and phosphatidylcholine (18:0/18:2) (PC (18:0/18:2)) were significantly upregulated, while triglyceride (26:4/29:4) (TG (26:4/29:4)) and phosphatidylcholine (46:14CHO) (PC (46:14CHO)) were notably downregulated. These lipids were primarily associated with the regulation of lipolysis in adipocytes and glycerolipid metabolism pathways. Proteomic analysis revealed 79 differentially expressed proteins. Post-race, proteasome subunits (alpha type_2, alpha type_5 isoform X1, alpha type_6, and beta type_2), carboxypeptidase E, and S-phase kinase-associated protein 1 showed significant downregulation. These proteins were primarily involved in the cellular catabolic process (Gene Ontology term) and pathways related to the proteasome and type I diabetes mellitus (Kyoto Encyclopedia of Genes and Genomes terms). Correlation analysis indicated a significant positive correlation between proteasome subunits (alpha type_2 and beta type_2) and PC (18:0/18:2), while a significant negative correlation was found with PC (46:14CHO). Conversely, S-phase kinase-associated protein 1, along with proteasome subunits (alpha type_5 isoform X1 and alpha type_6), exhibited a significant negative correlation with PE (18:0/16:0) and a positive correlation with TG (26:4/29:4). In conclusion, Yili horses may sustain energy balance and physiological equilibrium during racing by suppressing protein degradation and optimizing lipid metabolism. The differentially expressed substances identified could serve as key biomarkers for assessing exercise load in horses.

## Simple Summary

With the thriving development of equestrianism, unraveling the metabolic regulation mechanisms in high-intensity sports for horses has become a research hotspot aimed at enhancing racing performance, optimizing training programs, and preventing athletic injuries. In this study, we collected blood samples from the top three Yili horse participants who took part in pre- and post-5000 m racing. By conducting lipidomics and proteomics analysis, we identified 10 differential lipids and 79 differential proteins between the two groups, which significantly accumulated in pathways related to adipocyte lipolysis regulation, glycerolipid metabolism, proteasome, and type II diabetes mellitus. Our findings indicate that Yili horses may sustain their energy and physiological balance during the race by inhibiting protein degradation and optimizing lipid metabolism, thereby providing a theoretical basis for enhancing equine racing performance.

## 1. Introduction

The Yili horse, a renowned Chinese sport horse breed, descends from the Kazakh horse [1], with its breeding history spanning nearly a century. To address diverse production demands, the breed is categorized into several distinct groups, with the Yili racehorses primarily bred through crossbreeding between Thoroughbreds and Kazakh horses [2]. The rapid advancement of modern competitive horse racing has made the metabolic regulation mechanisms of horses during high-intensity exercise a key focus of research [3]. Its main objectives are to improve athletic performance, optimize training programs, and prevent injuries [4]. As a “dynamic carrier” of internal homeostasis, the plasma proteome and lipidome fluctuations in response to exercise provide comprehensive insights into physiological adaptations to stress, particularly in pathways like energy metabolism [5,6], oxidative stress [7,8], and inflammation [9,10]. Nevertheless, systematic molecular markers specific to post-race responses in Yili horses remain underexplored [11], and traditional methods relying on single physiological indicators fail to fully capture the dynamic nature of these responses [12].

After intense physical exertion, muscle contraction induces significant alterations in the body’s physiological state and metabolism. Studies conducted on both humans and mice demonstrate that strenuous exercise can lead to a temporary elevation in creatine kinase [13,14], lactate dehydrogenase [15,16], and proinflammatory markers such as IL-6 [17,18], as a result of muscle micro-injuries. Concurrently, free fatty acids increase during the early phases of exercise, driven by enhanced lipolysis [19,20,21]. Similar observations have been reported in equine research. For instance, post-exercise plasma levels of energy metabolism-related proteins, including apolipoproteins and AMP-activated protein kinase, are significantly elevated in horses [22,23]. Furthermore, the dynamic modulation of lipid molecules [24,25] plays a critical role in energy provision and cellular membrane integrity. However, most studies have concentrated on exercise simulations in controlled environments, such as training [26] or treadmill settings [27], with limited exploration of metabolic regulation in horses during actual competitive conditions. Additionally, while the unique genetic composition of the Yili horse may contribute to metabolic traits distinct from Thoroughbred or other breeds [28], research in this area remains scarce, impeding advancements in scientific training and precision breeding.

This study employed the Yili horse (5000 m racing) as the model to examine the dynamic alterations in plasma protein expression and lipid metabolism before and after exercise through non-targeted proteomics and lipidomics. The goal was to identify biomarkers associated with intense exercise-induced stress in the Yili horse, investigate the regulation of pathways related to energy metabolism and oxidative damage repair, and elucidate the metabolic mechanisms underlying exercise adaptability in this breed. The findings offer a theoretical foundation for enhancing equine sports performance, developing fatigue recovery strategies, and addressing metabolic disorders.

## 2. Materials and Methods

### 2.1. Ethics Statement

This study adhered to the Declaration of Helsinki and was approved by the Ethics Committee of Xinjiang Agricultural University (protocol code: 2024003).

### 2.2. Animals and Samples

This study involved 24 Yili horses that participated in a 5000 m race. These horses underwent specialized training by horse owners prior to the race, with eligibility confirmed only after a veterinarian verified their pedigree and health status. Blood samples were collected from the left jugular vein of all participating horses at 20:00 on the day before the race using Ethylene Diamine Tetraacetic Acid anticoagulation vacuum tubes. A total of 24 horses participated in our race. All of these horses have similar genetic backgrounds and they have been professionally trained by their owners in the same way as Thoroughbreds. Additional blood samples were collected from the top three horses immediately following the race to form the post-race group (Group A), and pre-race samples were found from these three horses before the race to form the pre-race group (Group B). The final race times of the three horses included in the final study were 5′23″704–5′41″339. All three horses are 4-year-old stallions. The blood samples were then centrifuged at 1000× *g* for 10 min to isolate plasma, which was subsequently frozen in liquid nitrogen and stored at −80 °C for subsequent lipidomics and proteomics analyses.

### 2.3. Lipid Extraction

The method described by Perera [29] was employed. Briefly, 100 µL of plasma was transferred into glass centrifuge tubes, with the addition of 0.75 mL of pre-cooled methanol. The tubes were vortexed thoroughly at room temperature. Next, 2.5 mL of pre-cooled methyl tert-butyl ether was added, vortexed, and 10 µL of SPLASH™ (Merck KGaA, Darmstadt, Germany) internal standard was introduced. The tubes were incubated for 1 h, after which 0.625 mL water (Mass Spectrometry-grade) was added, and the tubes were incubated for 10 min. The samples were centrifuged (1000× *g*, 10 min) to collect the upper organic phase. To the lower phase, 1 mL of extract (methyl tert-butyl ether/methanol/water in a 10:3:2.5 ratio, *v*/*v*/*v*) was added, and the organic phase was subsequently separated. The two organic phases were combined and concentrated under nitrogen, then reconstituted with 100 µL of isopropanol for analysis by Liquid Chromatography–Mass Spectrometry.

### 2.4. Liquid Chromatography–Mass Spectrometry

The procedure followed Liu’s method [30]. Specifically, samples were analyzed by Liquid Chromatography–Mass Spectrometry using a Thermo VanquishTM UHPLC chromatograph (Thermo Fisher Scientific Inc., Waltham, MA, USA) and a QExactiveTM HFX mass spectrometer (Thermo Fisher Scientific Inc., Waltham, MA, USA). Lipid separation utilized Thermo Accucore C30 column (Length × Diameter: 150 mm × 2.1 mm, Particle size: 2.6 µm) at 40 °C, with a flow rate of 0.35 mL/min and a gradient elution over 20 min. Mobile phase A consisted of acetonitrile/water (6:4) with 10 mM ammonium acetate and 0.1% formic acid, while buffer B was acetonitrile/isopropanol (1:9) with 10 mM ammonium acetate and 0.1% formic acid. The gradient program was as follows: 0–2 min, 30% mobile phase B; 2–5 min, 43% mobile phase B; 5–11 min, 55% mobile phase B; 11–18 min, 99% mobile phase B; 18–20 min, 30% B.

The mass spectrometer operated in both positive and negative ion modes, covering an *m*/*z* range of 114–1700. The automatic gain control target was set to 3 × 10^6^, with a maximum injection time of 100 ms. The normalized collision energies were set at 22 eV, 24 eV, and 28 eV [22 eV, 24 eV, 28 eV].

### 2.5. Lipidome Data Processing and Analysis

Raw data were processed using Lipidsearch 5.1 (Thermo Fisher Scientific, Waltham, MA, USA) for tasks such as peak identification, extraction, and secondary lipid identification. The primary parameters included a parent ion mass deviation of 5 ppm and a daughter ion mass deviation of 5 ppm. Subsequent qualitative and quantitative analyses were conducted based on the peak data. Principal Component Analysis (PCA) and Partial Least Squares Discriminant Analysis (PLS-DA) were performed using MetaboAnalyst 5.0 software to assess the similarity and difference of the samples within the group and to generate VIP values for each metabolite. Differential lipids were selected based on Variable Importance in the Projection (VIP) > 1, Fold Change (FC) > 1.2 or FC < 0.833, and *p* < 0.05. Lipid compounds were annotated with functional classifications, referencing key databases such as Kyoto Encyclopedia of Genes and Genomes (KEGG), Human Metabolome Database, and LIPID MAPS. Volcano plots were generated using the R package ggplot2, and cluster heat maps were created using Pheatmap. Based on the quantitative changes in lipid compounds, Gene Set Enrichment Analysis (GSEA) was performed for KEGG pathways.

### 2.6. Proteomics Sample Preparation

Plasma samples underwent depletion of high-abundance proteins using the ProteoMiner™ Protein Enrichment Large-Capacity Kit (1633007, BIO-RAD, Hercules, CA, USA). Subsequently, 500 µL of plasma was mixed with urea buffer (8M urea, 100 mM TEAB, pH 8.5), vortexed for 30 s, and 1 M Dithiothreitol was added for a 1 h incubation at 56 °C. Following a 2 min ice bath, Iodoacetamide was introduced and incubated in the dark for 1 h. Protein concentration was determined using the Bradford protein assay kit (Beyotime, Shanghai, China). Tetraethylammonium bromide (50 mM) and trypsin (1 µg/µL) were sequentially added to the filtrate. Enzymatic digestion was carried out at 37 °C for 4 h, followed by overnight digestion with additional trypsin and CaCl_2_. The sample was then centrifuged at 12,000× *g* for 5 min, and peptides were desalted using a C18 column (Evosep, Odense, Denmark). The final protein extract was lyophilized to obtain freeze-dried powder.

### 2.7. Data-Independent Acquisition Mass Spectrometry Detection

All samples were analyzed using the Vanquish™ Neo UHPLC-Astral Liquid Chromatography–Mass Spectrometry Data-independent (Thermo Fisher Scientific Inc., Waltham, MA, USA) acquisition method, with 10 µL of mobile phase A to dissolve the protein powder. The liquid phase parameters were as follows: the liquid chromatography system was the Vanquish™ Neo UHPLC (Thermo Fisher, Thermo Fisher Scientific Inc., Waltham, MA, USA); mobile phase A included 100% water with 0.1% formic acid, and mobile phase B contained 80% acetonitrile with 0.1% formic acid. Chromatographic conditions included the following: the C18 pre-column used was 174500 (5 mm × 300 µm, 5 µm, Thermo Fisher, Thermo Fisher Scientific Inc., Waltham, MA, USA), and the C18 analytical column was ES906 (PepMap™ Neo UHPLC, 150 µm × 15 cm, 2 µm, Thermo). The column temperature was 40 °C. The elution gradient was as follows: 0–0.2 min, flow rate 2.5 µL/min, 4% mobile phase B; 0.2–0.3 min, flow rate 2.0 µL/min, 4% B; 0.3–7.5 min, flow rate 1.5 µL/min, 8% B; 7.5–12.2 min, flow rate 1.5 µL/min, 22.5% B; 12.2–12.6 min, flow rate 2.5 µL/min, 55% B; 12.6–13.0 min, column wash; 13.0–13.7 min, flow rate 2.5 µL/min, 99% B. The Thermo Orbitrap Astral mass spectrometer (Thermo Fisher Scientific Inc., Waltham, MA, USA)with an Easy-Spray ion source was used. The primary mass spectrometer full scan range (*m*/*z*) was set to 380–980 with a resolution of 60,000 and automatic gain control set to 500%. The secondary *m*/*z* acquisition range was 150–2000, with a product ion resolution of 80,000 and a maximum injection time of 3 ms. The normalized collision energy was set to 25%.

### 2.8. Proteomics Data Analysis

The detected data were processed using DIA-NN software (V1.8) to extract chromatographic peak information for qualitative and quantitative peptide analysis. Protein quantification results were analyzed statistically using the *t*-test, with differential proteins identified based on FC > 1.2 or FC < 0.833 and a *p*-value < 0.05. Functional annotation was conducted with InterProScan software (5.72-97.0) for Gene Ontology (GO) and Interpro, and DAPs were subjected to volcano plot analysis, cluster heat map analysis, and enrichment analysis of GO, Interpro, and KEGG pathways.

### 2.9. Correlation Analysis Between DALs and DAPs

The Pearson correlation coefficient (r2) and *p* value between DALs and DAPs were calculated, and a correlation heatmap was generated. Differential metabolites were considered significantly correlated with differential genes when *p* < 0.05, as indicated by an asterisk (*) in the Figure.

## 3. Results

### 3.1. Lipidomics Detection and Identification of Differential Lipids (DALs)

**Figure 1:**
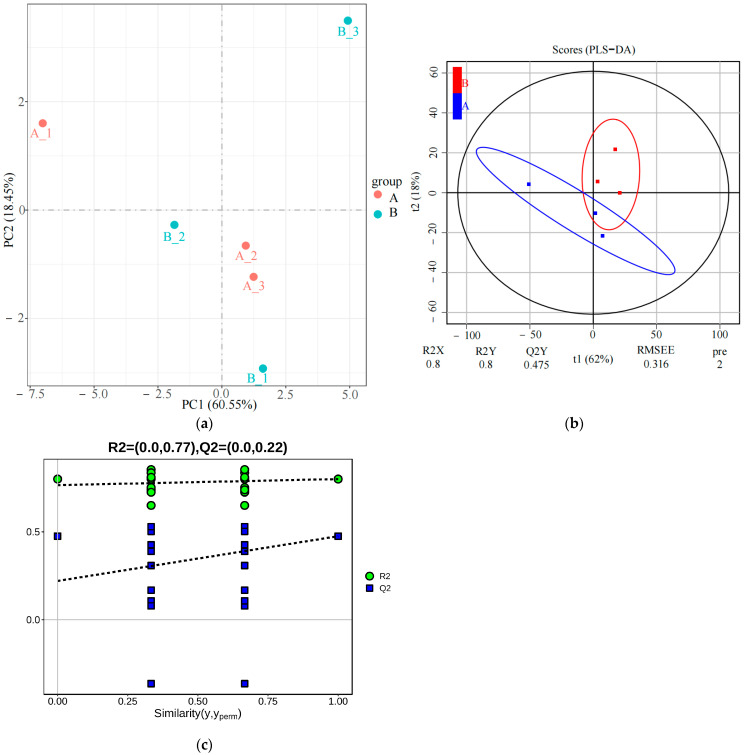
Comparison of serum lipid profiles among Yili horses. (**a**) PCA score; (**b**) PLS-DA score; (**c**) PLS-DA permutation test. R2 represents the proportion of data variance or dispersion that the current model can explain, while Q2 reflects the proportion of data variance that the current model can predict.

This study identified and quantified 724 distinct lipid metabolites. PCA of the lipid metabolites from the two sample groups revealed that the PC1 (first principal component) accounted for 60.55% of the variance, while the PC2 explained 18.45% (Figure 1a). PLS-DA confirmed the separation between the two groups, with an R2Y value of 0.800 (Figure 1b). The permutation test showed an R2 value of 0.77 and a Q2 value of 0.22, demonstrating the stability and reliability of the PLS-DA model (Figure 1c). These results indicate a shift in the plasma lipid metabolism pattern before and after a 5000 m race.

**Figure 2:**
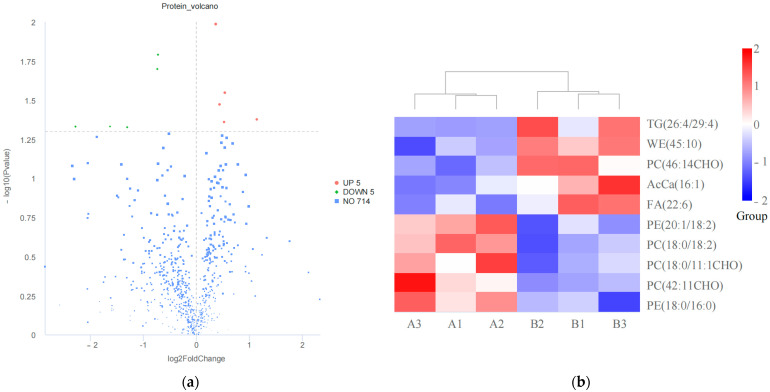
Pre- and post-race lipidomics features of Yili horse. (**a**) Volcano plot of differential lipid compounds; (**b**) cluster heatmap of differential lipid compounds.

In this study, VIP > 1, FC > 1.2 or FC < 0.833, and *p* < 0.05 were established as the criteria for differential screening. A total of 10 differential lipids (DALs) were identified between the two sample groups (Table S1, Figure 2a). Compared to pre-race samples, five lipids were significantly upregulated, while five were significantly downregulated in post-race samples. These DALs were classified into six subclasses: Phosphatidylcholine (PC), Phosphatidylethanolamine (PE), Fatty Acids (FA), Wax Ester (WE), Triglycerides (TG), and Acyl CoAs (AcCa). Hierarchical clustering analysis revealed distinct patterns between the two groups, indicating differential expression of DALs across the sample sets (Figure 2b).

### 3.2. Lipid Metabolism Pathways

**Figure 3:**
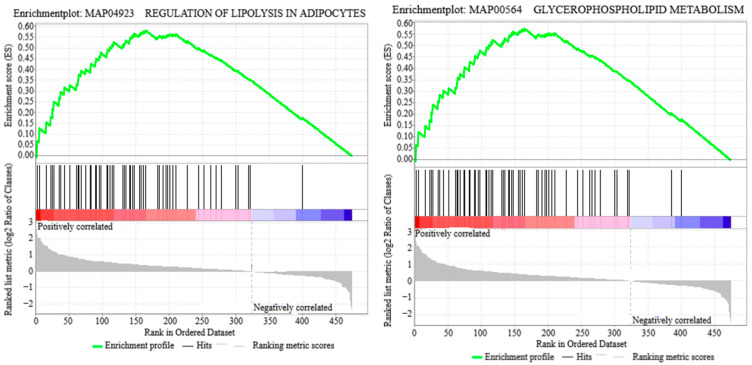

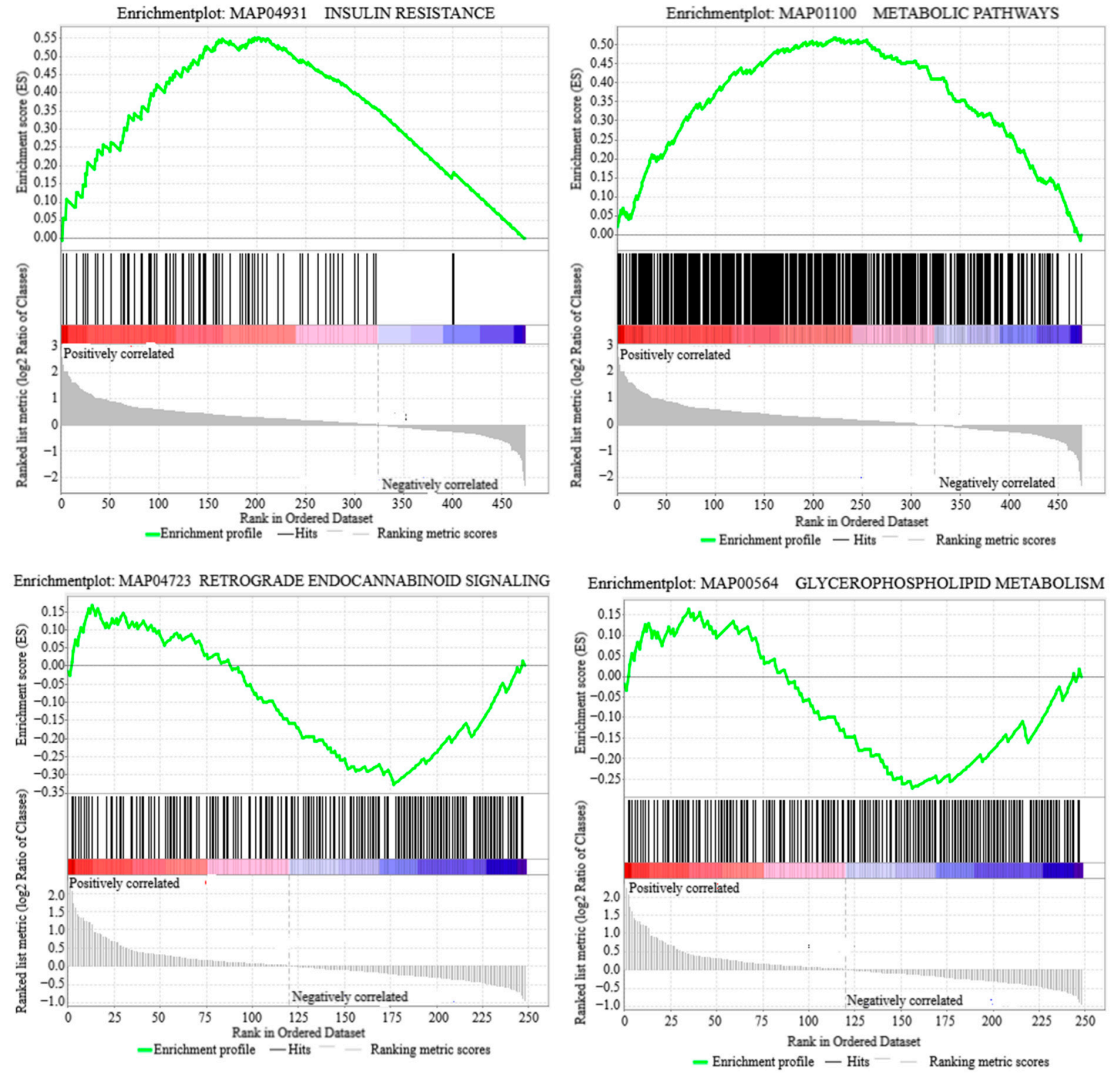
GSEA enrichment map.

GSEA analysis of the detected DALs identified several significantly enriched pathways, as summarized in Table S2 and Figure 3. These pathways included Regulation of Lipolysis in Adipocytes, Glycerolipid Metabolism, Insulin Resistance, Metabolic Pathways, Retrograde Endocannabinoid Signaling, and Glycerophospholipid Metabolism. The predominant enriched DALs within these pathways comprised TG (26:4/29:4), PC (46:14CHO), PC (18:0/18:2), PE (20:1/18:2), PE (18:0/16:0), and PC (18:0/11:1CHO).

### 3.3. Proteomics Detection and Identification of Differential Proteins (DAPs)

**Figure 4:**
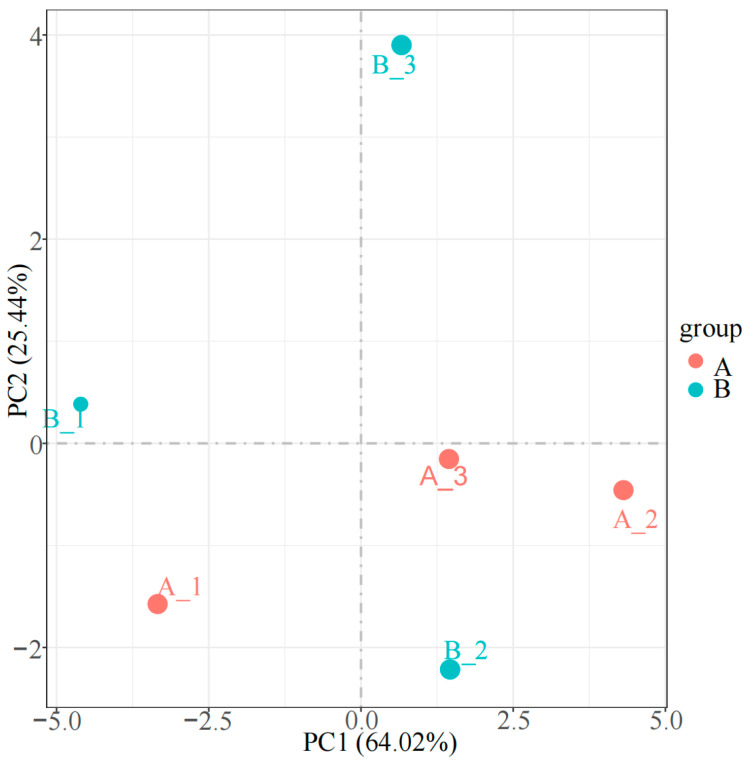
PCA analysis of the serum proteome in Yili horse before and after race.

The serum samples collected from the 5000 m pre-race and post-race phases were analyzed using DIA proteomics, resulting in the identification of 4084 peptides and 960 proteins. PCA revealed distinct differences in the proteomes of the two sample groups (Figure 4), with PC1 and PC2 accounting for 64.02% and 25.44% of the variance, respectively. The separation of proteins between the groups on the PCA plot suggested that the physiological states of the horses before and after the race differed significantly.

**Figure 5:**
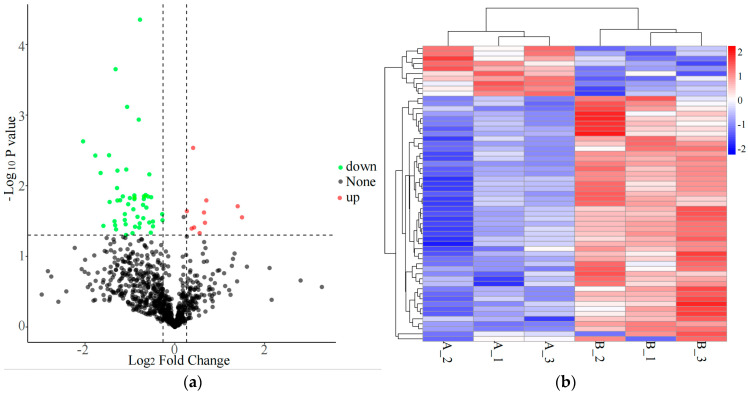

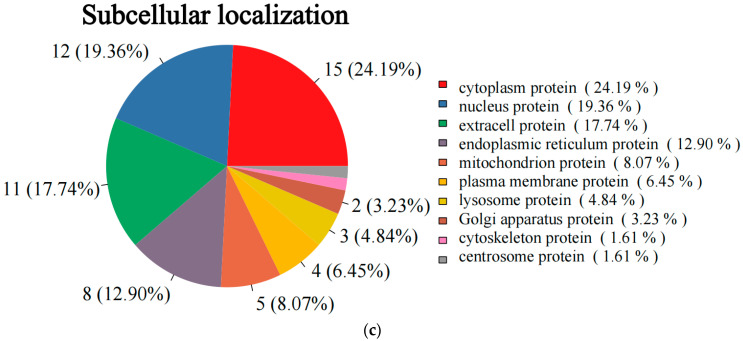
Identification of differential proteins. (**a**) DAPs volcano plot; (**b**) DAPs cluster map; (**c**) pie chart of DAPs subcellular localization annotation result.

This study identified 79 differential proteins (DAPs) by screening samples from both groups using criteria of FC > 1.2 or FC < 0.833 and *p* < 0.05. Among these, 12 DAPs were significantly upregulated in the post-race group (2 of which were undetected in the pre-race group), while 67 DAPs were downregulated (18 of which were undetected in the post-race group) (Figure 5a). A detailed list of these proteins was provided in Table S3. Cluster analysis of the DAPs revealed a clear hierarchical distinction between the two sample groups (Figure 5b). Subcellular localization analysis indicated that 62 DAPs were localized in various subcellular compartments, with the majority found in the cytoplasm (24.19%), nucleus (19.35%), and extracellular space (17.74%) (Figure 5c).

### 3.4. Enrichment Analysis of DAPs

**Figure 6:**
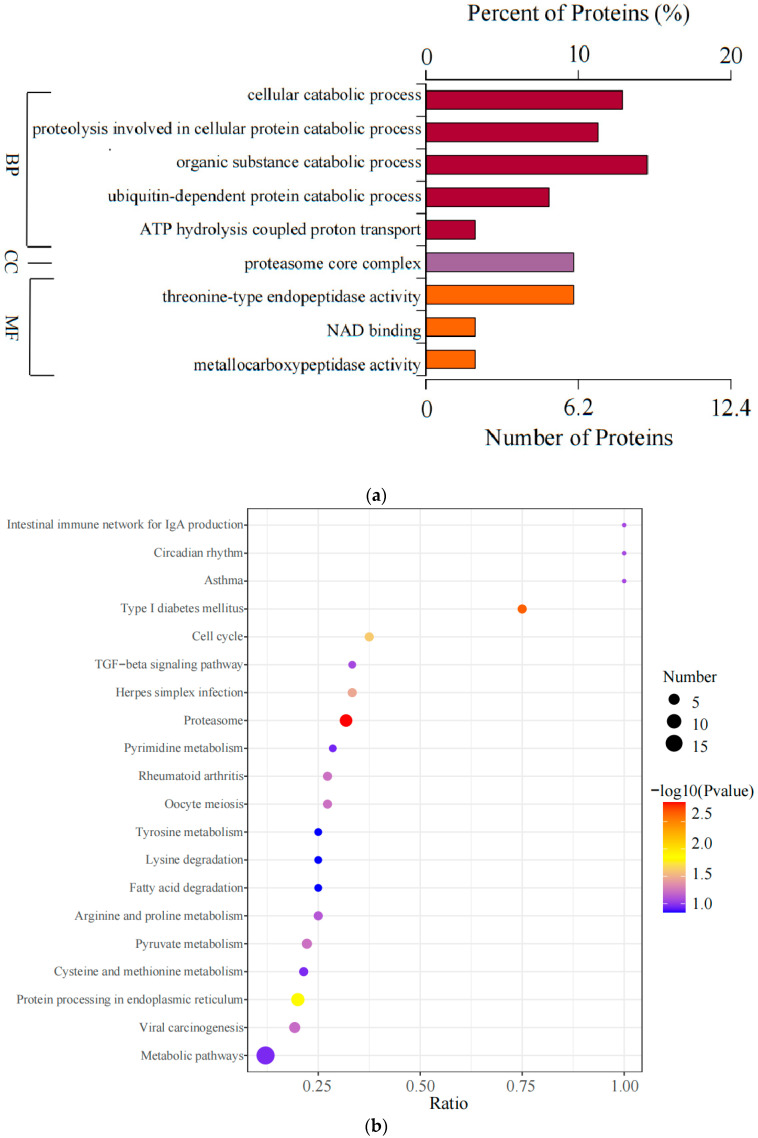
Enrichment analysis of DAPs. (**a**) Bar chart of GO enrichment analysis; (**b**) bubble chart of KEGG enrichment.

GO enrichment analysis (Figure 6a and Table S4) revealed that the identified DAPs were significantly associated with nine GO terms (*p* < 0.05), including organic substance catabolic process, cellular catabolic process, proteolysis involved in cellular protein catabolic process, threonine-type endopeptidase activity, and proteasome core complex, which contained the highest number of DAPs. These nine GO terms were categorized into BP, MF, and CC. KEGG pathway analysis further assessed the functional roles of the identified DAPs, highlighting significant enrichment in five pathways (Figure 6b and Table S5): Proteasome, Type I diabetes mellitus, Protein processing in the endoplasmic reticulum, Cell cycle, and Herpes simplex infection. Eight DAPs were enriched in both GO and KEGG pathways, specifically proteasome subunit alpha type_5 isoform X1, proteasome subunit beta type_7 isoform X1, proteasome subunit beta type_2, proteasome subunit alpha type_1, proteasome subunit alpha type_6, carboxypeptidase E, proteasome subunit alpha type_2, and S-phase kinase-associated protein 1. These DAPs were predominantly enriched in the cellular catabolic process (GO) and the Proteasome and Type I diabetes mellitus pathways (KEGG).

### 3.5. Correlation Analysis Between DALs and DAPs

**Figure 7:**
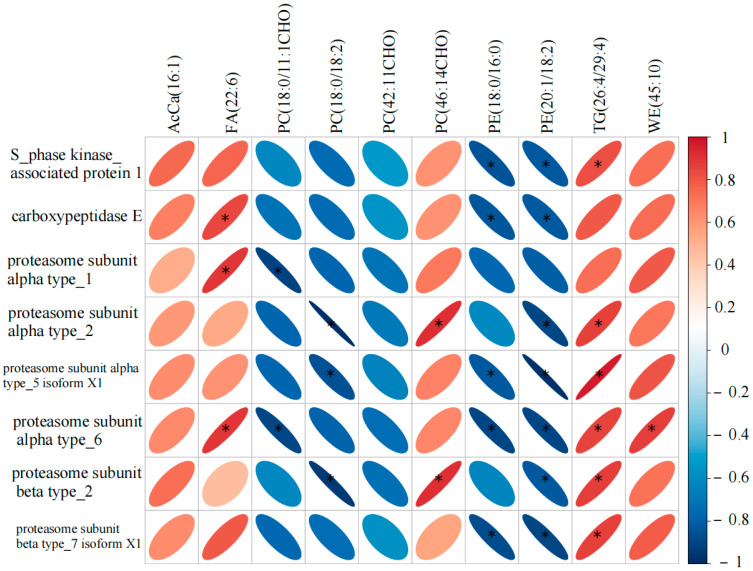
Heatmap of the correlation between DAPs and DALs. In the graph, blue indicates a negative correlation, and red indicates a positive correlation. The rounder the circle, the greater the Pearson correlation coefficient. * indicates *p* < 0.05.

The correlation analysis between the identified DALs and the eight principal DAPs is presented in Figure 7. Proteasome subunit alpha and beta type_2 exhibited a positive correlation with PC (18:0/18:2) and a significant negative correlation with PC (46:14CHO) (*p* < 0.05). S-phase kinase-associated protein 1, proteasome subunit alpha type_5 isoform X1, proteasome subunit alpha type_6, and proteasome subunit beta type_7 isoform X1 showed a negative correlation with PE (18:0/16:0) and a positive correlation with TG (26:4/29:4) (*p* < 0.05).

## 4. Discussion

High-intensity exercise could activate the sympathetic nervous system [31], enhance the activity of hormone-sensitive lipase and adipose triglyceride lipase in adipose tissue [32], and accelerate the hydrolysis of TG into free fatty acids and glycerol [33,34], thereby providing energy substrates for skeletal muscle contraction. Lipidomics analysis of pre- and post-race plasma samples from Yili horses revealed a significant reduction in TG (26:4/29:4) levels in post-race plasma, indicating that increased lipolysis and energy demand during high-intensity exercise promote fatty acid release [35]. TG (26:4/29:4) was notably enriched in the Regulation of lipolysis in adipocytes pathway, further demonstrating its role in meeting the elevated energy demands during exercise by activating the lipolysis pathway [36]. Phosphatidylcholine, a critical component of lipid metabolism and signal transduction, plays a significant role during exercise [37]. During intense physical exertion, the body’s energy demands surge, and the composition of fatty acids (including the proportion of polyunsaturated fatty acids) influences the fluidity of cell membranes and the efficiency of the electron transport chain [38], optimizing oxidative phosphorylation efficiency [39,40], thereby enhancing fat oxidation and meeting the body’s metabolic energy needs [41]. In this study, PC (18:0/18:2) exhibited a significant increase post-race, likely due to its role in enhancing signal transduction through improved membrane localization [42,43]. Exercise triggers lipolysis, releasing free fatty acids into the bloodstream [44]. Saturated fatty acids such as palmitic acid (16:0) and stearic acid (18:0) may be preferentially re-esterified to form PE (18:0/16:0), serving as a reserve for energy storage or membrane repair. The post-exercise increase in PE (18:0/16:0) highlights its role in optimizing lipid metabolism during physical exertion. Phosphatidylcholine exhibits substantial resistance to oxidative damage [45]. A significant reduction in PC (46:14CHO) following exercise suggests its potential in preserving membrane integrity by limiting lipid peroxidation. The significant enrichment of PC (18:0/18:2), PE (18:0/16:0), and PC (46:14CHO) in the Glycerophospholipid metabolism pathway highlights the central role of glycerophospholipid remodeling in the cellular adaptive response to exercise, ensuring both energy metabolism and membrane stability [46,47].

In this study, multiple proteasome subunit isoforms (e.g., alpha type_2, alpha type_5 isoform X1, alpha type_6, and beta type_2) were significantly downregulated post-race, suggesting that the ubiquitin-proteasome system (UPS) activity is likely inhibited following intense exercise [48,49]. This response may reflect a temporary regulation in which protease activity is suppressed in the short term as part of the body’s adaptive reaction to oxidative stress. Overactivation of proteases could lead to excessive degradation of functionally important proteins, while transient inhibition could allow for a protective window to facilitate cellular repair [50]. Additionally, this inhibition might trigger compensatory activation of the autophagy pathway, which has been shown to clear damaged proteins when protease function is impaired [51,52]. The downregulation of various proteasome subunit isoforms in Yili horses post-race suggests that this mechanism helps coordinate protein metabolism pathways to optimize cellular repair after exercise. Furthermore, the major DAPs in the proteasome pathway were all downregulated, indicating a “functional inhibition” of the pathway post-exercise. This may serve as a protective mechanism to prevent excessive protein degradation and muscle dysfunction, while also redirecting energy toward cellular repair processes [53].

As a prohormone convertase, downregulation of carboxypeptidase E may regulate the biosynthesis of hormones such as insulin and neuropeptides [54]. Reduced carboxypeptidase E levels in the plasma of post-race Yili horses may impair insulin production, thereby limiting glucose consumption during exercise and preventing excessive declines in blood glucose levels. Additionally, carboxypeptidase E participates in stress response mechanisms; its downregulation could mitigate oxidative damage and preserve cellular stability by decreasing protein synthesis related to cellular stress [55]. S-phase kinase-associated protein 1, a key component of the Skp, Cullin, F-box containing complex, was significantly downregulated post-race [56]. This reduction may delay cell proliferation, allowing for the prioritization of damaged cell repair through decreased degradation of cell cycle inhibitors [57]. Furthermore, Skp, Cullin, F-box containing complex plays a role in activating proinflammatory factors [58,59], and the downregulation of S-phase kinase-associated protein 1 may contribute to the modulation of the inflammatory response post-exercise.

The correlation between differential lipids and differential proteins observed between the two groups of samples can be used as evidence of metabolic pathway remodeling [60]. The correlation observed in our study between specific proteasome subunits and multiple lipids suggests a potential regulatory crosstalk between protein metabolism and lipid metabolism during equine racing to maintain the body’s energy balance during exercise [61]. In our study, we found that Proteasome subunit (alpha and beta type_2) was positively correlated with PC (18:0/18:2), and Phosphatidylcholine is an important component of cell membranes, which is closely related to the permeability and mobile phase of cell membranes [62], which suggests that Proteasome subunit (alpha and beta type_2) has a regulatory role in maintaining cellular homeostasis.PE is mainly synthesized in mitochondria and then translocated to other cells [63]. We found that some Proteasome subunits (e.g., alpha type_5 isoform X1, alpha type_6) are associated with PE (18:0/16:0), suggesting that some subunits of the Proteasome subunit may have an effect on lipid storage and mobilization during exercise to maintain the energy supply of horses during competition. Taken together, our findings suggest that horses may maintain the balance of cellular structure and energy during competition through the mutual regulation of proteins and lipids. However, our study only sampled the top three horses in the race, which may have an impact on our findings.

## 5. Conclusions

This study demonstrates that post-race Yili horses modulate protein degradation in stages and enhance glycerophospholipid metabolism by downregulating PC (46:14CHO), TG (26:4/29:4), proteasome subunits (alpha type_2, alpha type_5 isoform X1, alpha type_6, and beta type_2), carboxypeptidase E, and S-phase kinase-associated protein 1, while upregulating PC (18:0/18:2) and PE (18:0/16:0). This regulatory network may optimize energy distribution, mitigate oxidative damage, and improve repair efficiency. Such a distinctive balance in protein and lipid homeostasis likely molecularly underpins the horse’s exceptional athletic performance and suggests that these differential components in plasma could serve as potential biomarkers for monitoring exercise load.

## Data Availability

The data used and analyzed during the current study are available from the corresponding author upon reasonable request.

## Supplementary Materials

The following supporting information can be downloaded at: https://www.mdpi.com/article/10.3390/ani15070994/s1, Table S1: Basic Information of Differential Lipid Metabolites; Table S2: GSEA Analysis of Differential Lipid Metabolites; Table S3: Basic Information of Differential Proteins; Table S4: GO Enrichment Analysis of Differential Proteins; Table S5: KEGG Enrichment Analysis of Differential Proteins.
